# Abscess in the Substance of the Brain; the Lateral Ventricles Opened by an Operation

**Published:** 1850-02

**Authors:** 


*Abscess in the Substance of the Brain; the Lateral Ventricles opened by rn Operation.* By Wm. Detmold, M. D. of New York.

Francis Miller, ætat. 40, of a rather phlegmatic temperament, a well developed head, and good constitution, a native of Germany, was, on the morning of the 14th of July, 1849, at work in a machine shop, when a derrick broke down, and a piece of the machinery struck him with great force on the left side of the os frontis. The force of the blow may be estimated from the fact that the same piece which struck Miller killed two men, besides severely injuring some others, who were all at work underneath.

Miller was carried home insensible, and was first seen by two medical men, whose names I have not ascertained, and who ordered cold applications. My friend Dr. Miller was called in in the evening, and found the patient fully recovered from his insensibility, the wound and the parts around much swollen, the conjunctiva of the left eye much congested. There being no very urgent symptoms, and the swelling precluding a minute examination of the wound, Dr. Miller ordered the cold fomentations to be continued; bled the patient freely, and prescribed a solution of the nitrate of potash, and a strictly antiphlogistic regimen. Under this treatment. the patient got along very well; the wound yielded a healthy pus, and the swelling gradually went down. About the third week after the injury, a bloody diarrhœa came on, the patient having had an attack of dysentery before the accident occurred. This relapse yielded readily to proper treatment.

On the 8th of August, Dr. Miller discovered a loose piece of bone in the wound, which he removed, and which measured in its longest diameter, which was the transverse one, a full inch, and in its short diameter onethird of an inch; the piece consisted of the entire thickness of the frontal bone, which was remarkably thin. After the removal of the first piece, Dr. Miller, upon careful examination, found another piece of bone loose, but was unable to remove it without dilating the wound. Fearing that this piece of bone, if left in its position, might lead to trouble, he requested me to see the patient the following day with him.

On the 9th of August, I saw the patient for the first time; I found a small suppurating wound on the left side of the os frontis, about three inches above the supra-orbitar margin, and about midway between the linea semicircularis and the median line of the os frontis. The patient was lying in bed, with slight symptoms of pressure on the brain. He was in a state of apathy rather than of stupor; he could easily be roused, and answered all questions promptly; his pulse natural, about 75 per minute, without any farther morbid symptoms. I found the loose piece of bone, which Dr. Miller had described to me, at the upper edge of the wound, in which the denuded dura mater could be felt pulsating. I dilated the wound upwards, and discovered that the piece of bone in question was only a few lines in circumference, but that the fracture extended farther in the inner table of the bone than the outer, which was perfectly firm. With a strong forceps, and making horizontal traction, so as to avoid pressure and perhaps laceration of the dura mater by the inner edge of the bone, I succeeded with a slight effort in disengaging the piece, which measured nearly a square inch of the tabula interna. During the enlarging of the external wound, the patient exhibited great sensitiveness, and immediately after the removal of the piece of bone, he brightened up, and professed himself decidedly relieved. Upon further examination with the finger, I did not discover any more loose bone; and, the patient declaring himself so decidedly better, and very averse to farther interference, which indeed at the time did not appear to be called for, cold applications and strictly antiphlogistic regimen were ordered, and I left the patient again under charge of my friend Dr. Miller, with the remark that I feared more trouble.

In a few days, fresh and healthy granulations sprung up in the wound, which in about a fortnight was entirely closed, and the patient went about again apparently well, and without complaining of anything.

On the morning of the 13th of September, nearly nine weeks after the accident, and about five weeks after the removal of the above mentioned pieces of bone, Dr. Miller called on me again, and stated that the patient had, on the day previous, complained of headache, and had become drowsy. He had seen the patient just before he called on me, about nine o'clock in the morning, and had found him in a stupor, from which however, he could be roused, and, when requested to show his tongue, would readily put it out: pulse 60 in the minute. As I had to operate for stone in the bladder at one o'clock, P. M., I invited Dr. Miller to be present, immediately after which, I promised to see his patient. I invited all the gentlemen who witnessed the operation for stone, also to see this case. Prof. S. H. Dickson, Dr. Carnochan, Dr. Miller, Dr. Schirmer, Dr. Wilhelm, Dr. Michaelis, Dr. Meyer, and several others whose names I do not at present recollect, besides some ten or twelve medical students, accompanied me to witness the following operation. It was about two o'clock before all the above gentlemen reached the house, which was at a distant part of the city.

We found things much changed for the worse, since Dr. Miller had seen the patient in the morning. The patient was now in a profound stupor, from which nothing could rouse him, lying motionless, but neither paralyzed nor convulsed; breathing stertorous; pulse 40 in a minute, and occasionally intermitting; pupils immovable. In the place of the original wound, we found a deep adhering cicatrix, which communicated to the finger distinctly the pulsation of the brain.

There was evidently no time to be lost; but we had first to overcome the most obstinate resistance on the part of the family and friends of the patient. Considering him hopeless and dying, they were unwilling to have his last moments disturbed. As I afterwards learned, nothing but the large number of doctors present induced them to yield. We labored under the disadvantage of not having things as well prepared as we could have wished, for, fearing the opposition might gain strength, we lost but little time in preparations. Thus, we had a decidedly bad and insufficient light, and I do not know whether I should have been able to succeed, had I not been ingeniously aided by Prof. Dickson, who, by means of a looking-glass threw a full reflected light upon the field of operation.

The time which had elapsed since the accident, in addition to the fact that the patient had been about until lately, although, according to his wife's account, he had not been so lively since the accident, left, under the present symptoms, very little doubt as to the nature of the case. I anticipated an abscess, and even thought that I felt fluctuations under the adhering scar. I stated this before commencing the operation, but admitted that I was not positive, being aware that the tense dura mater, with the brain underneath, might deceive me.

I commenced by surrounding the adhering cicatrix with an incision, so as to leave that at first untouched, and then made several incisions in different directions, in search for fractured bone. In the upward direction, where I had on the former occasion removed the piece of bone, I found the skull firm; but the fracture extended inward towards the median line, downward towards the supra-orbitar ridge, and outward towards the semicircular ridge; so that the original wound, and the first deficiency of bone, formed the upper and middle point of the entire wound. By carefully carrying on the incisions, as far as I could readily with my finger separate the pericranium from the bone, I successively removed three large pieces of bone, which, including some small plinters, and the two pieces removed before, made an opening into the left half of the os frontis which measured about five square inches. The opening extended to within half an inch of the supra-orbitar margin; and the outer piece had formed part of the linea semicircularis. All the pieces showed strong marks of absorption, the edges being rounded off, and the surface uneven and porous. Professor Dickson expressed his fear that the supra-orbitar margin might be fractured, and the injury extend to the base of the skull—a fear which the final result proved to be but too well founded. But, as all the edges which surrounded this now large defect of bone were apparently firm and immovable, and as the pericranium everywhere strongly adhered to the bone, I concluded that I had removed all the fragments, although the condition of the patient was not in the slightest degree improved. By the lateral incision, I had divided the temporal artery, and allowed it to bleed freely, but it made no impression on the patient. It was remarkable, and I looked upon the fact as a favorable sign, that, notwithstanding his profonnd stupor and general insensibility, the patient exhibited unmistakable signs of intense pain at every incision through the integuments. After having cleared the wound of blood, the dura mater appeared, throughout the whole extent of the wound, in a natural condition, with the exception of the adhering cicatrix, which had been left as a small island in the upper part of the wound. Finding that the removal of the fragments of bone had not changed the condition of the patient, and also finding all the bony limits of the defect firm and without any depression, I proceeded to dissect off the adhering cicatrix, removing with it the dura mater to the same extent, as the cicatrix was adhering to it, and brought the brain itself into view, covered with the pia mater, which appeared thickened. I then introduced a p;obe under the dura mater, and passed it around the whole opening between the dura and pia mater, to ascertain whether anything morbid could be found there; but the probe passed freely round, being plainly visible through the transparent membrane. I now felt again with great anxiety, for the fluctuction which I thought I had detected before commencing the operation; but the removal of such a large portion of bone, and the opening of the dura mater itself, had taken away all tension, and I did not discover any fluctuation. Confident, however, that an abscess in some part of the brain was the cause of the present condition of the patient, and equally certain that, unless speedy relief was given, he must inevitably sink within a few hours, I determined to make an incision into the brain; and, in the hope that my first impression about the fluctuation had been correct, and that, as I stated above, the removal of the tension had rendered the fluctuation indistinct again, I chose this place, where the brain was now already denuded of dura mater, and made an incision into the substance of the brain, about one inch in length and about half an inch in depth, which was followed instantaneously by a thick stream of healthy looking pus; further discharge was then favored by gentle pressure on the dura mater, and by proper position of the head. I regret that, at the time, no means were taken to ascertain the exact quantity of the matter discharged; but the circumstances, and the uncertainty of finding the matter, will easily account for that omission. The quantity was afterwards variously estimated by the different medical gentlemen present; several estimated it as high as five ounces, while the lowest estimate exceeded two ounces. The effect of the discharge was almost magical; for the patient immediately opened his eyes, put out his tongue when requested, and answered distinctly that he felt better, the pulse rose immediately to above 60 per minute. The wound was covered with warm water dressing, and the head placed as much as possible in a position to facilitate the discharge. Absolute rest and water gruel were recommended.

The patient passed a quiet night, enjoying sound sleep; and awoke, comfortable and refreshed, next morning. The wound continued to discharge freely, and, in a few days, all the incisions healed, leaving merely an opening to the extent to which the original scar, with the corresponding portion of the dura mater, had been removed. A probe introduced into the opening passed into a cavity of about three inches in diameter, extending nearly towards the median line. On account of a tendency of the opening to close, and cause the cavity to fill again with matter, a small tent of lint was introduced, and the warm water dressings continued. The patient was in every respect doing well; sat up in bed at every dressing of the wound; conversed freely; was cheerful; and had a good appetite. Therefore, no farther interference, with the exception of a simple enema every third day, was deemed necessary: and, taking into consideration the length of time which had elapsed since the accident; the comparatively slight suffering of the patient while carrying about this collection of matter; the immediate and complete relief which the opening and discharge of the abscess had given him—together with the fact that the anterior lobe of the brain was the seat of the injury—I was sanguine in my prognosis, in spite of my friend Professor Dickson, who took a different view of the case.

About the tenth day after the operation, the brain began to protrude where it was exposed, and, on the twelfth day, the hernia cerebri had reached the size of a large walnut. As the discharge from the abscess had considerably diminished, I determined to apply moderate pressure, which was effected in the following manner: I covered the protruding portion of the brain with lint, over which I applied thick compresses, which were held firmly down by broad strips of muslin, glued to the shaved head with collodion. The patient bore this amount of pressure well, and the discharge found its way through the dressing. Three days after, I removed the dressing, and found that the hernia cerebri had receded to a level with the integuments, showing a rosy and healthy granulating surfaee. The wound was now covered with a large and rather heavy warm poultice, which was kept in place by an elastic nightcap, making still a slight pressure; and the danger from hernia cerebri seemed to be past. The exposed part of the brain began to cicatrize, and the patient was able to leave his bed about the eighteenth day after the opening of the abscess. Still, there was a small opening, which allowed the probe to pass into a cavity in the anterior lobe of the brain; this cavity did not seem to show any inclination to fill up entirely, although the whole place where the bone was removed was deeply drawn in.

About three weeks after the operation, although the patient was out of bed the greater part of the time, and, to the inquiry how he felt, answered generally "first rate," he began to lose his memory. This amnesia increased rapidly; so that, in a few days, he did not recollect either his own name or the names of his wife or children. When asked the name of his wife, he frequently answered Catherine, the name of his long-deceased mother; but, when assisted by giving him the first two syllable, "E-li," he smiled, and readily answered Elizabeth. He recognized me, but did not recollect my name or my business. When I asked him whether I was a blacksmith or a shoemaker, or the like, he smiled, and said "No." When I asked whether I was the doctor, he readily replied "Yes, the doctor." He would frequently, when asked a question, instead of answering, simply repeat the two or three last words of the question. It was remarkable to observe the attempt to recollect things, which he would generally give up with a desponding shake of the head. Although he seemed to have forgotten all names, he recognized and recollected the name and value of money. Occasionally, however, he would call a dollar, hundred cents, as though the word dollar had escaped his memory; yet he remembered the value, and substituted that,—a train of ideas which appears hardly compatible with such a complete loss of memory. A strange fact occurred about this time, of which, however, I was not an eye-witness. It was communicated to me by Dr. Miller. The weather getting cold, a stove was put up in the room; and he tried to explain to his brother that he had something at the workshop which he wished to be brought home; but he did not recollect the name of the thing. He then took a piece of chalk, and tried to make himself understood by drawing the object. He at last succeeded in drawing a shovel, and, while thus engaged, he suddenly recollected the word, and described accurately the place where they would find it in the shop. At first, he wrote his name with comparative ease, although, when asked, he did not recollect it; soon after, when asked again to write it, he attempted it, but did not succeed, although the letters were told him. He was also able at first to read, but soon lost that accomplishment, though not entirely, for, on days when he was brightest, he would still be able to read a few words. After having been asked several questions, and attempted to recollect things, his countenance assumed an expression of deep distress, evidently the result of mental fatigue, combined with the painful consciousness of loss of memory. His mental faculties were not always equal, but varied from day to day; they were always brightest immediately after the dressing of the wound and the emptying of the abscess.

He contined in this condition till the 18th of October. The place where the bone had been removed had sunk in considerably, encouraging me in the hope that the cavity might close by the walls being thus brought close together: but about that time the place again began to become more prominent; the skin felt hot, and was red; the patient was unwilling to leave his bed, and had an attack which, according to his wife's description, must have been something like a convulsive tremor; he also complained of headache. On the 22d of October, the parts had become very prominent, and the patient was in a condition approaching to stupor. As the probe could not be passed to the same depth as formerly, I concluded that the opening of the cavity had closed, and that a new accumulation of matter had taken place. I therefore made another incision through the integuments into the brain, to the depth of one and a quarter inch, but without finding any pus. On this occasion, Professors Dickson and Granville S. Pattison were present. There being no very urgent symptoms, these gentlemen agreed with me that it would not be prudent, for the present, to risk cutting deeper, as the incision which I had made must have come very near the abscess, and as the matter might probably discharge spontaneously into the incision. But I was much astonished to find the patient, the next day, much better. His memory even was brighter than it had been for several days before this last incision, and the protrusion had sunk in, although, upon the closest examination, I could not discover that any matter had been discharged. On introducing the probe, it passed, to my utter astonishment, to a depth of four and a half inches into the brain, taking the direction of the lateral sinus, into which, evidently, the abscess had opened. Several medical men, among whom I may mention Dr. Gurdon Buck, Dr. Reese, Dr. Ashbell Smith of Texas, saw me pass the probe to that distance into the brain, and coincided with me in opinion that it intered the lateral sinus. From tnat time, the patient again began to mend bodily; but he soon lost his memory entirely, though able to be part of the day out of bed. His appetite was good; he slept well; and did not complain. On the 26th of October, he had another rigor; he passed his urine involuntarily; vomited; and the outer walls of the cavity again commenced to protrude. On the 27th of October, he was speechless; but would nod his head when asked a question, and put out his tongue when requested. He evinced intense pain upon the slightest pressure of the abdomen. On the 28th, all these symptoms had increased; and it was evident that the scene was drawing to a close. I was, however, unwilling to give up the case without making one more effort; and, having come to the conviction that the present state was caused by an accumulation of matter in the lateral sinus, I determined to make an incision into the lateral sinus; although, even if I succeeded in relieving the brain, the effusion, which, from the tenderness of the abdomen, I feared had taken place in the peritoneal cavity, must prove fatal.

About 12 o'clock, on the 28th of October, about sixteen weeks after the accident, and about seven weeks after I opened the first abscess, in presence of Dr. Dickson, Dr. Miller, Dr. Ayres, Dr. Michaelis, Dr. Palmedo, and others, I made an incision through the most prominent part of the integuments, penetrating fully an inch and a half into the substance of the brain, which appeared unchanged; but no pus followed the incision, altho' I thought on withdrawing the knife, that its point was stained by pus. I then introduced a probe, which passed readily into the lateral sinus four and three-quarter inches deep; but, not finding any pus, I abandoned further interference. The patient having passed no urine in twenty-four hours, I introduced a catheter, but found the bladder empty. I now considered that I had gone as far as I could possibly justify; and we left the patient to his fate. We had been gone scarcely five minutes, when matter began to flow freely from the wound; and the nurse caught about ℥ss of it in a glass. I went to see the patient again about 7 o'clock in the evening of the same day, and, just at the moment I entered the room, he calmly, and without a struggle breathed his last. I was, of course, anxious to make an examination, but gained, with the greatest difficulty, the permission from the friends to examine merely the head on the same evening. I called upon Prof. Dickson, Prof. Pattison, Dr. Bowen, and Dr. Miller; and, at my request, Prof. Pattison himself had the kindness to make the post-mortem, two hours after death.

After removing the upper part of the skull, all the edges of the fractured bone were found rounded off by absorption, the dura mater in a congested state, the substance of the brain exhibiting on a cut surface rather more and larger red spots than normal; and, upon cutting down to the ventricles, both lateral ventricles were filled with a thin pus, the right ventricle containing a larger quantity than the left, which had partly discharged its contents before death through the wound. In the roof of the anterior corner of the left ventricle, a fresh incised wound was found, showing that I had, in the morning, carried my incision, as intended, into the ventricle. The uniting of the cut surfaces of the substance of the brain, immediately after the passage of the knife through it, had prevented the matter from being directly discharged. The septum pellucidum was broken down by suppuration. There was a deposite of lymph intermixed with pus upon the choroid plexus, and the same deposit could be traced through the third and fourth ventricle, making its appearance at the base. The supra-orbitar ridge was broken, and the whole left orbitar plate of the frontal bone was broken into small fragments. The abdomen was not opened.

This case has evidently many points of interest, as well to the surgeon as to the physiologist and phrenologist. I abstain from all remarks upon the case, as all I might say will readily suggest itself to the reader.—*Am. Jour. of Medical Sciences.*

[We omit the remainder of the article, which consists of the record of the case made by Prof. Dickson in his private note-book, and is merely a repetition of the facts already presented.—Ed. Buf. Med. Jour.

